# Association of institutional volume with surgical and oncologic outcomes for robotic rectal cancer surgery: a retrospective review

**DOI:** 10.1007/s00464-026-13234-1

**Published:** 2026-08-06

**Authors:** Mohamed H. El-Farra, Liang Ji, Jonathan Lee, Jenny Cevallos, Marc Abou Assali, David Row, Sharon Lum, Raja R. Narayan, Rhami Khorfan

**Affiliations:** Department of General Surgery, Loma Linda University, Loma Linda, CA USA

**Keywords:** Proctectomy, Robotic, Oncologic, Volumes

## Abstract

**Introduction:**

Robotic surgery has been increasingly adopted for rectal cancer. However, the majority of studies investigating robotic surgery have focused on high-volume institutions. This study aims to compare the perioperative and oncologic outcomes of robotic rectal cancer surgery at low-, medium-, and high-volume institutions.

**Methods:**

Adults undergoing robotic proctectomy for primary rectal cancer were identified from the National Cancer Database (2010–2022). Institutions reporting ≥ 2 years of cases were included. A generalized additive model was used to determine volume thresholds classifying hospitals into low, medium, and high volume based on their rates of conversion to open surgery. Patient characteristics and outcomes were evaluated with chi-squared analysis. Overall survival was assessed with multivariable Cox proportional hazards modeling.

**Results:**

Among 327 institutions, thresholds for low, medium, and high were 1–4, 5–13, and > 13 cases/year. The total number of patients and facilities for low, medium, and high were 10,945/142, 17,361/147, and 8,828/38, respectively. High-volume institutions (HVIs) operated more frequently on cN1-2 tumors (46.0, 50.0, 53.5%, *p* < 0.0005), but fewer poorly differentiated tumors (10.2, 9.8, 8.4%, *p* < 0.0003). HVIs had lower rates of conversion to open (7.9, 5.2, 3.2%, *p* < 0.0001), lower 90-day mortality (2.2, 1.4, 1.3, *p* < 0.0001), and more frequently resected ≥ 12 lymph nodes (76.8, 80.5, 85.7%, *p* < 0.0001). On multivariable analysis, there was no difference in long-term overall survival based on institutional volume (medium vs. low: HR 0.97 [0.88–1.07], *p* = 0.52; high vs. low: HR 0.92 [0.82–1.03], *p* = 0.13).

**Conclusions:**

Differences in patient selection exist for robotic rectal cancer resection based on hospital volume. HVIs demonstrated better oncologic and perioperative outcomes, but there was no difference in long-term overall survival based on volume. There is an opportunity for low- and medium-volume institutions to achieve appropriate long-term oncologic outcomes, but further work is needed to investigate patient selection and early post-operative mortality.

The surgical management of rectal cancer is defined by its anatomical complexity, requiring precise total mesorectal excision (TME) within the restrictive confines of the bony pelvis [1, 2]. In recent years, the surgical landscape has undergone a paradigm shift, characterized by an increase in the usage of robotic platforms for these operations [3]. Data from national registries indicate that for complex resections such as low anterior resections (LAR), the adoption of robotic technology has risen consistently as surgeons seek to utilize its advanced technical capabilities [1].

Despite this rapid integration into standard oncologic practice, the current understanding of robotic surgical outcomes is heavily skewed. Most existing evidence is derived from high volume, specialized tertiary institutions where robotic programs are mature and standardized [4]. In these high-volume settings, surgeons have typically surpassed the established learning curve allowing for optimized operative times and refined perioperative protocols [5, 6].

Consequently, there is a lack of clinical data representing outcomes from low-volume institutions. While current data reflects appropriate safety and oncologic outcomes, it remains unclear if these results are reproducible in settings with lower annual case volumes where multidisciplinary infrastructure may differ [1, 2, 7]. Investigating the “volume-outcome” relationship across a broader national landscape is therefore essential to ensure that the technical benefits of the robotic platform translate into consistent safety and oncologic quality for all patients, regardless of hospital size. Therefore, this study aims to compare the perioperative and oncologic outcomes of robotic rectal cancer surgery at low-, medium-, and high-volume institutions.

## Methodology

The 2022 participant user file of the National Cancer Database (NCDB) was used in this analysis. The NCDB is a hospital-based tumor registry, run jointly by the Commission on Cancer of the American College of Surgeons Commission and the American Cancer Society, which captures approximately 70% of all newly diagnosed cancer cases in the US. The database contains de-identified information on patient demographics, tumor staging, treatment, and survival.

Patients with primary rectal cancer undergoing low anterior resection (LAR) or abdominoperineal resection (APR) between 2010 and 2022 were considered for inclusion in this analysis. Patients were excluded if they were less than 18 years of age at the time of diagnosis, if their indication for surgery was not for primary rectal malignancy, or if they did not undergo a robotic LAR or APR. Robotic cases that converted to open were also included in this study. Institutions with fewer than two years of reported data were also excluded so that only hospitals who are now past the early learning phase of robotic surgery were included in this study. This study was granted exempt status by our Institutional Review Board.

Patient-level data included in the analysis consisted of age, race, ethnicity, sex, insurance status, and comorbidity data in the form of a modified Charlson comorbidity score. Hospital-level data included hospital type and median years reporting cases. Tumor-specific data included histologic subtype, clinical stage, tumor size, tumor grade, and nodal status. Treatment-specific data included operation type (LAR vs. APR with or without colorectal or coloanal anastomosis), days from diagnosis to surgery, and receipt of adjuvant or neoadjuvant therapy. Outcomes included median length of stay, 30-day readmission, 30-day mortality, 90-day mortality, rate of conversion to open, lymph node yield, and post-operative margin status.

To estimate the association between institutional volume and conversion to open, a generalized additive model with restricted cubic splines was used to specify the functional form of the volume thresholds. This methodology provides a flexible model for non-linear relationships between a continuous predictor (e.g., surgical volume) and the rates of a certain outcome (e.g., conversion to open surgery), without assuming a fixed functional form. The model suggests threshold values that represent inflection points in the rate of conversion to open. In this way, the cutoff values for low-, medium-, and high-volume categories were informed by the data itself, and not arbitrary volume thresholds. Statistical comparison of patient characteristics and outcomes was performed using chi-squared analysis. Long-term overall survival was assessed via Kaplan–Meier analysis and multivariable Cox proportional hazards modeling, adjusting for clinically relevant covariates. Inverse probability of treatment weighting was applied for all multivariable adjusted analyses in order to mitigate selection bias and control for measured confounding.

This study was reported in accordance with the Strengthening the Reporting of Observational Studies in Epidemiology (STROBE) guidelines.

## Results

**Fig. 1:**
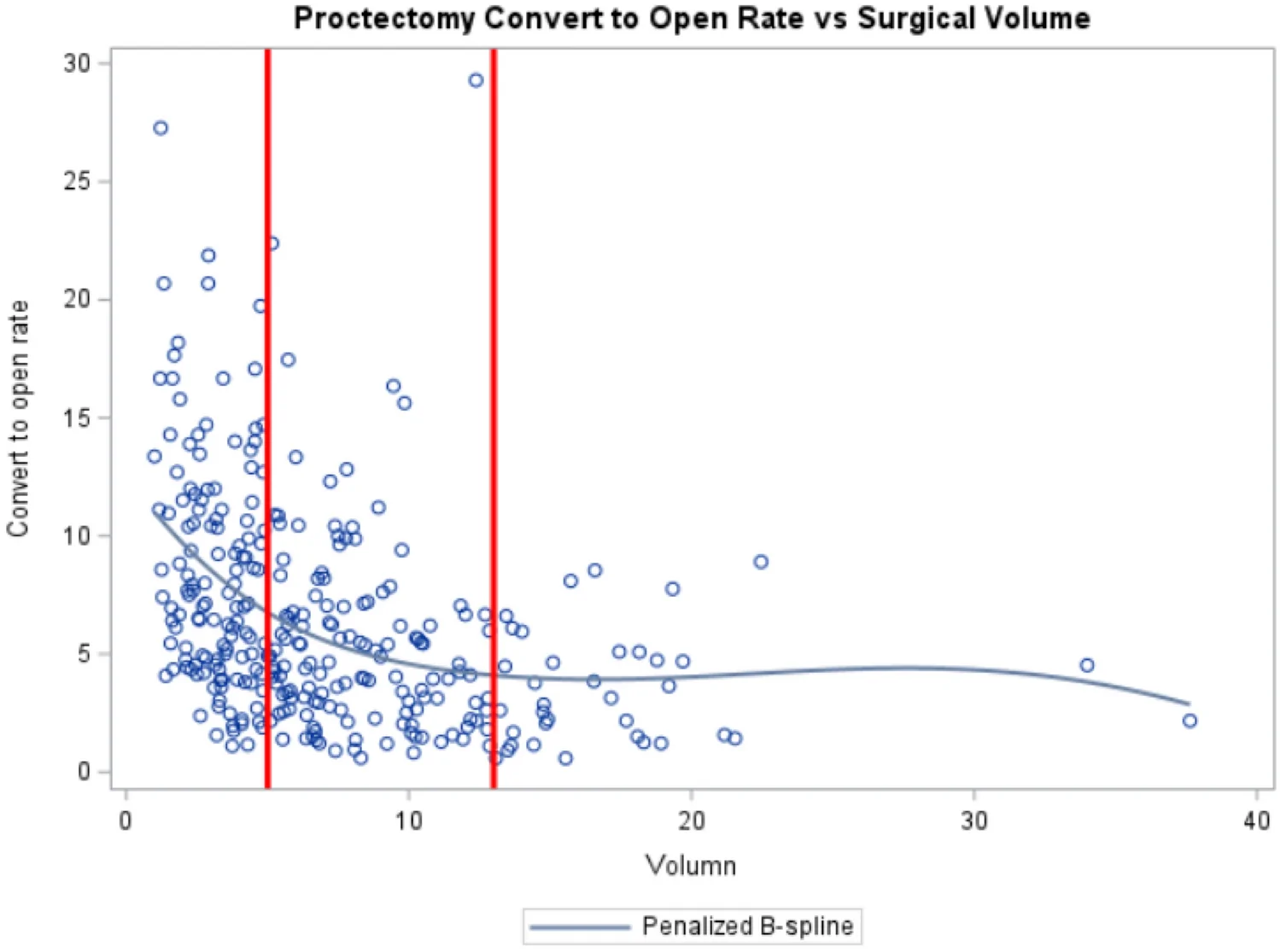
Volume threshold cutoffs determined by generalized additive model

The final cohort consisted of 37,134 patients treated across 327 institutions. Based on the generalized additive model, institutional volume thresholds were determined to be low (1–4 cases per year), medium (5–13 cases per year), and high (> 13 cases per year) (Fig. 1). High-volume institutions (HVIs) generally operated on younger patients (median age 59 [50–67] vs. 61 [52–70] in low-volume centers, *p* < 0.0001) with lower Charlson–Deyo score ≥ 2 (6.2 vs. 7.5%, *p* < 0.0042). HVIs were more frequently academic centers compared to low-volume institutions 60.1 vs. 24.2%, *p* < 0.0001) (Table 1).

**Table 1:** Analysis of patient and hospital characteristics

	Low-volume (≤ 4 cases/y) *n* = 10,945	Medium-volume (5–13 cases/y) *n* = 17,361	High-volume (≥ 13 cases/y) *n* = 8,828	*p*-value
Median age (IQR), years	61 (52–70)	60 (51–69)	59 (50–67)	** < 0.0001**
Male Sex	6,793 (62.1%)	10,752 (61.9%)	5,468 (61.9%)	0.8453
Ethnicity (Non-Hispanic White)	8,059 (75.9%)	13,369 (78.5%)	6,946 (80.8%)	** < 0.0001**
Charlson–Deyo score ≥ 2	819 (7.5%)	1,310 (7.6)	550 (6.2%)	**0.0042**
Insurance status (private)	5,122 (47.3%)	9,057 (52.6%)	5,116 (58.6%)	** < 0.0001**
Academic facility	2548 (24.2%)	5633 (34.2%)	4956 (60.1%)	** < 0.0001**
Median years of reporting (IQR)	9 (6–10)	11 (9–13)	13 (11–13)	** < 0.0001**

*IQR* interquartile range

Bold data represents *p*-value < 0.01

Analysis of various oncologic factors revealed there was no difference in clinical T3–T4 staging (76.2 vs. 75.5%, *p* = 0.2405); however, HVIs more frequently operated on clinical nodal 1–2 stages (53.5 vs. 46.0%, *p* < 0.0005). Low-volume institutions operated more frequently on poorly differentiated tumors (10.2 vs. 8.4%, *p* = 0.0003) (Table 2). HVIs performed more low anterior resections with coloanal anastomosis than lower-volume centers (7.6 vs. 5.9%, *p* < 0.0001). Low-volume institutions more frequently gave neoadjuvant chemotherapy (30.9 vs. 29.0%, *p* = 0.0006) as well as adjuvant chemotherapy (16.0 vs. 12.9%, *p* < 0.0001) and adjuvant radiation (7.3 vs. 5.3%, *p* < 0.0001) (Table 3).

**Table 2:** Analysis of tumor characteristics

	Low-volume (≤ 4 cases/y) *n* = 10,945	Medium-volume (5–13 cases/y) *n* = 17,361	High-volume (≥ 13 cases/y) *n* = 8,828	*p*-value
Adenocarcinoma histologic subtype	10,691 (97.7%)	17,002 (98.0%)	8,647 (98.0%)	0.5692
Clinical T3-T4 stage	429 (75.5%)	11,015 (77.3%)	5,736 (76.2%)	0.2405
Tumor size mm (IQR)	40 (26–57)	40 (26–57)	41 (28–57)	0.0497
Clinical N1-2 stage	4,562 (46.0%)	7,939 (50.0%)	4,393 (53.5%)	**0.0005**
Tumor grade, poorly differentiated	905 (10.2%)	1,372 (9.8%)	623 (8.4%)	**0.0003**

*IQR* interquartile range

Bold data represents *p*-value < 0.01

**Table 3:** Analysis of treatment-specific data

	Low-volume (≤ 4 cases/y) *n* = 10,945	Medium-volume (5–13 cases/y) *n* = 17,361	High-volume (≥ 13 cases/y) *n* = 8,828	*p*-value
Low anterior resection with coloanal anastomosis	672 (6.1%)	1,024 (5.9%)	674 (7.6%)	** < 0.0001**
Median time from diagnosis to surgery (days)	136 (63–198)	143 (77–218)	147 (81–237)	** < 0.0001**
Neoadjuvant radiation	6,661 (62.0%)	11,069 (65.0%)	5,527 (63.4%)	0.0142
Neoadjuvant chemotherapy	3,359 (30.9%)	4,800 (27.9%)	2,543 (29.0%)	**0.0006**
Adjuvant radiation	784 (7.3%)	1,013 (6.0%)	460 (5.3%)	** < 0.0001**
Adjuvant chemotherapy	1,736 (16.0%)	2,308 (13.4%)	1,134 (12.9%)	** < 0.0001**

Bold data represents *p*-value < 0.01

Overall outcomes revealed HVIs demonstrated the lowest rates of conversion to open surgery (3.2 vs. 7.9% at low-volume centers, *p* < 0.0001). Furthermore, HVIs achieved significantly lower unadjusted 90-day mortality rates compared to low-volume centers (1.3 vs. 2.2%, *p* < 0.0001). When comparing oncologic outcomes, HVIs more frequently resected ≥ 12 lymph nodes (85.7 vs. 77.0%, *p* < 0.0001) and examined a higher number of regional lymph nodes (median 17 vs. 15, *p* < 0.0001). There was no difference in rate of positive circumferential resection margins between the different institutions (14.7 vs. 15.2 vs. 13.7%, *p* = 0.09) (Table 4).

**Table 4:** Analysis of perioperative and oncologic outcomes

	Low-volume (≤ 4 cases/y) *n* = 10,945	Medium-volume (5–13 cases/y) *n* = 17,361	High-volume (≥ 13 cases/y) *n* = 8,828	*p*-value
Median length of stay (days)	4 (3–7)	4 (3–6)	4 (3–6)	** < 0.0001**
30-day readmission	732 (6.7%)	1,156 (6.7%)	518 (5.9%)	0.0261
Unadjusted 30-day mortality	106 (1.1%)	102 (0.7%)	45 (0.6%)	** < 0.0001**
Unadjusted 90-day mortality	206 (2.2%)	209 (1.4%)	101 (1.3%)	** < 0.0001**
Conversion to open rate	861 (7.9%)	901 (5.2%)	282 (3.2%)	** < 0.0001**
Negative surgical margin	10,171 (93.8%)	16,283 (94.3%)	8,295 (94.4%)	0.0475
Median lymph nodes examined	15 (12–20)	16 (15–21)	17 (13–24)	** < 0.0001**
Lymph nodes resected (≥ 12)	8,190 (77.0%)	13,622 (80.5%)	7,391 (85.7%)	** < 0.0001**

Bold data represents *p*-value < 0.01

**Fig. 2:**
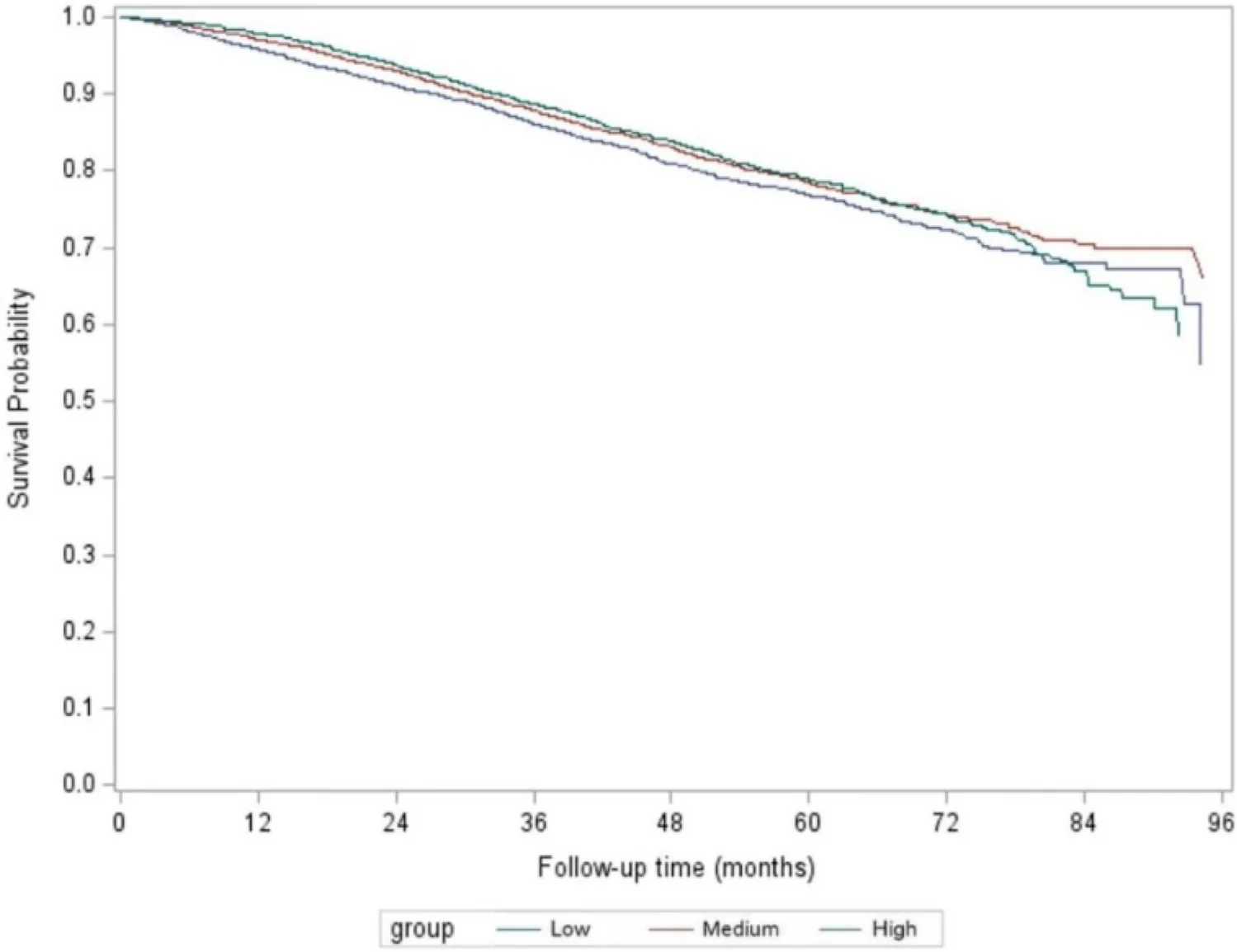
Kaplan–Meier survival curve (low- vs. medium- vs. high-volume institutions)

Multivariable analysis adjusted for patient, tumor, and treatment factors showed no significant difference in long-term overall survival based on institutional volume (Fig. 2) (medium vs. low: HR 0.97 [0.88–1.07], *p* = 0.52; high vs. low: HR 0.92 [0.82–0.1.03], *p* = 0.13) (Table 5).

**Table 5:** Multivariable analysis of outcomes and overall survival

Variable	Volume Comparison		95% CI Interval	*p*-value
		*Count Ratio*		
Length of stay	Medium vs. Low	0.96	0.94—0.97	** < 0.0001**
	High vs. Low	0.91	0.89—0.93	** < 0.0001**
Regional lymph nodes examined (≥ 12)	Medium vs. Low	1.07	1.05—1.08	** < 0.0001**
	High vs. Low	1.19	1.17—1.21	** < 0.0001**
		* Odds Ratio*		
Conversion to open	Medium vs. Low	0.65	0.59—0.71	** < 0.0001**
	High vs. Low	0.39	0.34—0.45	** < 0.0001**
30-day readmission	Medium vs. Low	1.02	0.92—1.12	0.7426
	High vs. Low	0.91	0.81—1.03	0.1452
Negative surgical margin	Medium vs. Low	0.94	0.84—1.04	0.2162
	High vs. Low	0.99	0.87—1.13	0.8874
		* Hazard Ratio*		
Overall survival	Medium vs. Low	0.97	0.88—1.07	0.5150
	High vs. Low	0.92	0.82—1.03	0.1290

Bold data represents *p*-value < 0.01

## Discussion

This study demonstrates that institutional volume is associated with differences in patient selection for robotic proctectomy, as well as some aspects of perioperative safety and short-term oncologic quality. Specifically, HVIs adopted a robotic approach in patients with less co-morbidities, but higher N1 and N2 disease. This requires further investigation but may reflect selection of patients with higher oncologic complexity, but less clinical risk. Additionally, patients treated at low-volume institutions (LVIs) more frequently received neoadjuvant chemotherapy as well as adjuvant chemotherapy and radiation. The relatively low rate of neoadjuvant chemotherapy overall, particularly at HVIs, can likely be explained by the time period of the study, which was largely prior to the widespread adoption of total neoadjuvant therapy [8, 9]. On the other hand, the difference in adjuvant therapy may be related to patient selection, as LVIs operated on more patients with poorly differentiated tumors. Perhaps reflecting some of this risk, LVIs demonstrated higher unadjusted short-term mortality. However, there were no differences in long-term overall survival based on institutional volume. The parity in long-term survival suggests that the oncologic efficacy of a successful robotic resection is consistent once the perioperative window is navigated. This finding is supported by matched-cohort data showing that robotic and laparoscopic resections provide similar survival rates despite the technical approach [1, 2, 10]. Meta-analyses have also confirmed that while robotics may reduce blood loss and hospital stays, pathologic outcomes like CRM positivity and lymph node harvest often remain equivalent to other approaches in balanced cohorts [3, 11].

The disparities in early outcomes may be linked to the institutional learning curve. Establishing a proficient robotic program is estimated to require approximately 75 cases, after which individual surgeons may reach proficiency in fewer cases [5]. High-volume environments likely benefit from matured multidisciplinary teams and standardized protocols, which have been shown to significantly reduce docking times and optimize post-operative recovery [4, 6]. However, this provides an opportunity for low- and medium-volume institutions to improve their outcomes to provide safe oncologic resections using the robotic platform.

Prior randomized trials have demonstrated no difference in outcomes for robotic vs. laparoscopic rectal cancer surgery [2, 7]. These were all performed in high-volume academic centers, with surgeons performing a large number of laparoscopic and robotic resections. These results may not be generalizable to the reality of surgery in the United States, where rectal cancer care is not concentrated at a handful of high-volume centers, and most rectal cancer surgery is done in the community by surgeons who perform a relatively lower volume of cases. While debate continues regarding the best minimally invasive approach, there is broad consensus that minimally invasive rectal surgery provides significant benefits compared to open surgery, regardless of the specific modality. It is also well established that the learning curve for robotic surgery is much shorter than laparoscopic surgery [12]. In this context, the robotic approach may therefore have broader acceptance and improved patient access in scenarios where the alternative would be open surgery. In reality, for the majority of surgeons, the decision is not one between a robotic or laparoscopic approach, but between an open and a minimally invasive approach. If the robotic platform offers a lower barrier of entry to minimally invasive rectal surgery, then more patients will have access to the benefits of minimally invasive surgery.

This study is subject to several limitations inherent to the use of the NCDB. While the NCDB is a reliable clinical registry containing data abstracted by trained and dedicated cancer registrars, there are some data fields that are not required for collection by every site. This can introduce a significant degree of missingness for some variables. Specifically, data regarding tumor size, tumor grade, and clinical T stage often contain gaps because they are derived from “site-specific” codes that are not required for submission by all participating centers.

Additionally, specific clinical details are unavailable in the NCDB, particularly details regarding the multidisciplinary care of cancer patients. These include the exact duration, type, and number of cycles for chemotherapy, and other details that are critical to the multimodal treatment of rectal cancer particularly in the era of total neoadjuvant therapy regimens. Additionally, the database does not consistently distinguish between upper, middle, and low rectal cancers, nor does it provide granular data on specific post-operative complications such as anastomotic leak, local recurrence, or the frequency of fecal diversion. To address these gaps, we utilized 30-day readmission as well as 30- and 90-day mortality as surrogate data points for major perioperative complications.

Furthermore, the NCDB does not permit the tracking of individual surgeons within an institution, which precludes the analysis of surgeon-level volume and variation in treatment and outcomes.

## Conclusion

In robotic rectal cancer surgery, high-volume institutions achieve superior perioperative results, including lower conversion rates and reduced 90-day mortality. However, institutional volume is not associated with differences in long-term overall survival. These findings suggest that while high-volume centers provide a safer "window" for immediate surgical care, lower-volume institutions can achieve appropriate long-term oncologic outcomes through refined patient selection and improved management of early post-operative mortality.
